# Assessment of Skin Autofluorescence and Its Association with Glycated Hemoglobin, Cardiovascular Risk Markers, and Concomitant Chronic Diseases in Children with Type 1 Diabetes

**DOI:** 10.3390/nu16121940

**Published:** 2024-06-19

**Authors:** Marta Jankowska, Agnieszka Szadkowska, Iwona Pietrzak, Jędrzej Chrzanowski, Julia Sołek, Wojciech Fendler, Beata Mianowska

**Affiliations:** 1 Department of Pediatrics, Diabetology, Endocrinology and Nephrology, Medical University of Lodz, 91-738 Lodz, Poland; marta.jankowska87@gmail.com (M.J.); agnieszka.szadkowska@umed.lodz.pl (A.S.); iwona.pietrzak@umed.lodz.pl (I.P.); 2 Department of Developmental Neurology and Epileptology, Polish Mother’s Memorial Hospital-Research Institute, 93-338 Lodz, Poland; 3 Department of Biostatistics and Translational Medicine, Medical University of Lodz, 92-215 Lodz, Poland; jedrzej.chrzanowski@umed.lodz.pl (J.C.); wojciech.fendler@umed.lodz.pl (W.F.); 4 Department of Pathology, Chair of Oncology, Medical University of Lodz, 92-213 Lodz, Poland; juliasolek@gmail.com

**Keywords:** skin autofluorescence, advanced glycation end products, type 1 diabetes, children, HbA1c, cardiovascular risk, body fat, celiac disease, diabetes complications

## Abstract

Skin autofluorescence (sAF) measurement is a non-invasive method used to assess tissue advanced glycation end product (AGE) accumulation. This study aims to characterize sAF’s association with (1) glycated hemoglobin (HbA1c) values, (2) cardiovascular risk markers, and (3) common comorbidities (autoimmune thyroiditis, celiac disease) in children with type 1 diabetes (T1D). Materials and methods: A total of 348 children with T1D aged 3–18 years and 85 age- and gender-matched control subjects were enrolled. sAF was quantified using an AGE Reader (Diagnoptics BV, The Netherlands). The analysis covered HbA1c, blood lipid, and C-reactive protein (CRP) levels, ambulatory blood pressure monitoring records, and body composition parameters. The associations between variables and sAF were assessed using the Mann–Whitney U test and Spearman correlation. Results: We observed significantly higher sAF values in the T1D group compared to the control (1.40 [1.27–1.53] vs. 1.20 [1.07–1.30, AU]; *p* = 0.004), consistent across all tested age groups. In the T1D group, sAF was positively correlated with current HbA1c, mean of historical HbA1c values, and T1D duration (r values, respectively: 0.27, 0.22, 0.14, all *p* < 0.01). Percentage of body fat was positively correlated with sAF (r = 0.120; *p* = 0.044). No significant correlations were found between sAF and lipid fractions, Z-score of BMI, parameters from 24 h ambulatory blood pressure monitoring, or the amount of albumin excreted in urine. sAF was positively correlated with CRP (r = 0.17, *p* < 0.05). sAF was significantly higher in patients with concomitant celiac disease (1.53 [1.43–1.63] vs. 1.40 [1.27–1.53, AU], *p* = 0.001). Conclusion: Among young T1D patients with relatively brief diabetes duration, sAF effectively mirrors prior glycemic control, as presented by historical average HbA1c. However, associations with conventional CV risk markers are not evident. The higher sAF values in patients with celiac disease warrant further exploration.

## 1. Introduction

Type 1 diabetes (T1D) is associated with the development of vascular damage, including changes in small blood vessels (microangiopathy, i.e., diabetic kidney disease, retinopathy, and neuropathy) and cardiovascular (CV) disease (macroangiopathy, i.e., coronary artery, peripheral artery, and cerebrovascular disease) [1,2]. The goal of treating children and adolescents with T1D is to prevent acute and chronic complications of the disease and to achieve the normal growth and development of the child while providing the best possible quality of life for the patient and their caregivers, similar to persons without T1D. To this end, children with T1D are often screened for microvascular and CV complications, including periodic evaluations of urinary albumin excretion, blood pressure values, and blood lipid levels, and ophthalmologic examination [3]. The clinical evaluation also includes regular (typically biannual) investigations of common T1D autoimmune comorbidities, such as celiac disease and autoimmune thyroiditis (especially Hashimoto’s thyroiditis) [4]. The key tenet of long-term diabetes management is the achievement of adequate metabolic control, which can be assessed by glycated hemoglobin A1c (HbA1c), a gold-standard biomarker of the long-term metabolic control of T1D and a validated biomarker of the development of micro- and macrovascular complications, for which the recommended target value for young people with diabetes has been set to less than 7.0% (<53 mmol/mol) [3,5]. Although HbA1c is of practical importance, this biomarker is unreliable in some cases (e.g., the coexistence of certain types of anemia) [6].

One of the mechanisms involved in the pathogenesis of chronic diabetes complications (microvascular and macrovascular) is the excessive production of advanced glycation end products (AGEs), including the glycation of collagen and proteins in tissues and body fluids [1]. AGEs can be measured in a skin biopsy (an invasive test) or via a skin autofluorescence measurement (sAF: a non-invasive method) [7]. In adults with T1D, sAF has been shown to reflect glycemic control, as assessed by HbA1c levels, and is associated with traditional CV risk markers [8,9,10,11,12].

Data about the utility of sAF measurement in children and adolescents with T1D as a proxy for diabetes control and to monitor the risk of CV complications have been inconclusive. Banser et al. showed a positive correlation between sAF and mean HbA1c concentration, total cholesterol, and triglyceride levels [13]. In turn, van der Heyden et al. demonstrated a lack of significant correlation between sAF and historical HbA1c values [14]. Neither of these papers directly addressed the application of sAF in assessing the risk of chronic diabetes complications, its risk factors (e.g., blood pressure values), or comorbidities with other common autoimmune diseases (e.g., Hashimoto’s or celiac disease). Only one study conducted on the adolescent T1D population (mean age: 15.6 years; diabetes duration: 8.7 years) demonstrated a positive correlation between sAF and the occurrence of retinopathy or autonomic neuropathy [15]. Values of sAF physiologically increase with age and might differ between ethnic groups. This indicates the desirability of defining and using age-specific reference ranges of sAF for individuals representing different populations [16,17,18].

The aim of this study was to evaluate in children and adolescents with T1D the association of sAF values with (1) glycemic control, assessed by current and mean long-term HbA1c levels, (2) CV risk markers and microvascular complications, and (3) the presence of concomitant diseases.

## 2. Materials and Methods

This is a cross-sectional study comprising patients aged 3–18 years with T1D of at least 6-month duration who were under the care of the Department of Pediatrics, Diabetology, Endocrinology and Nephrology at the University Pediatric Centre of the Central Clinical Hospital, Medical University of Lodz, Poland. Consecutive patients hospitalized for scheduled (typically biannual) diabetes-related re-education and re-evaluation (i.e., modifications of insulin therapy when necessary, or screening for diabetes-related complications and concomitant diseases) who fulfilled the inclusion criteria were included. Children with acute infections at the time of the study examinations were excluded. Age- and gender-matched control groups, including children without diabetes, were analyzed to assess the difference in sAF between cases and controls. For the control group, consecutive children referred for routine laboratory tests at the University Pediatric Centre central laboratory whose parents agreed for their child to participate in the study were included. The exclusion criteria for the control group were as follows: diagnosis of diabetes, symptoms characteristic of diabetes at the time of examination, renal failure, overt cardiovascular disease, or signs or symptoms of any acute illness at the time of examination [19]. The study protocol was approved by the Bioethics Committee of the Medical University of Lodz (approval RNN/49/18/KE, 15 February 2018). Written informed consent was obtained from participants’ legal guardians in addition to the participants’ assent. All investigations were conducted according to the principles of the Declaration of Helsinki.

Each participant’s glucose control was assessed via HbA1c levels from capillary blood, determined by high-performance liquid chromatography (HPLC) using the Variant Hemoglobin A1c Program (Bio-Rad Laboratories, Hercules, CA, USA; BioRad, Marnes-la-Coquette, France). For the case group, the mean of historical HbA1c values (from four months after diabetes diagnosis) was calculated. Each participant had their skin autofluorescence (sAF, in arbitrary units, AU) measured with the AGE Reader (Diagnoptics BV, Groningen, The Netherlands) on a clean skin area, free of skin lesions. During the measurement, a patient places the volar side of the forearm of the dominant limb on the base of the device, which, for several seconds, illuminates approximately 4 cm^2^ of the skin with light wavelengths of 300–420 nm. The sAF measurement result is calculated by the device as the ratio of the intensity of the emitted light (420–600 nm; total emission intensity) to the excitation intensity (300–420 nm), multiplied by 100 and given in arbitrary units (AU). Three independent measurements were performed for each patient at approximately 30 s intervals, and the average of three values was used as the final result. The mean relative error for the daily and seasonal intra-individual reproducibility of the sAF measurements performed with this technology for skin phototypes I-IV (low-to-medium skin pigmentation) was assessed to be 5% [20].

For the case group, traditional markers of CV risk, i.e., total cholesterol with its fractions (low-density lipoprotein (LDL) cholesterol and high-density lipoprotein (HDL) cholesterol) and triglycerides were measured (ARCHITEKT analyzer, Abbot, Wiesbaden, Germany), and ambulatory blood pressure monitoring values were recorded (ABPM) for patients 8 years and older (Spacelabs 90217 Monitor, Spacelabs, Issaquah, WA, USA). Similarly, all patients recorded anthropometric measurements with a body mass index (BMI) z-score, referenced to Polish pediatric centile charts [21]. We measured body composition (including the content of fat tissue) parameters (MC-980MA analyzer, TANITA, Tokyo, Japan) and the high-sensitivity blood levels of C-reactive protein (CRP) as a marker of the severity of systemic inflammation. Microvascular complication screening test results, i.e., urinary albumin excretion and the last ophthalmologic examination results, were also included in the analyses. Information on comorbidities, such as celiac disease and Hashimoto’s disease, was obtained from the patients’ medical records.

### Statistical Analysis

Continuous variables are summarized using the median with interquartile range (IQR) or mean with standard deviation (SD) based on the data normality determined by the Shapiro–Wilk test. Nominal variables are presented as the number of positive cases and the corresponding group percentage. To investigate the differences and correlations among the continuous variables, Student’s *t*-test, the Mann–Whitney U test, and Pearson or Spearman’s test were used, depending on the distribution of the variables. Calculations were performed using Statistica 13.3 software (TIBCO Software, Inc., Palo Alto, CA, USA). The study sample size was planned based on expected recruitment capabilities and the minimum required size for the detection of a difference between the case and control groups of sAF, with a standardized effect size of 0.3, set alpha of 0.05, statistical power of 0.8, two-sided two-group comparisons (Wilcoxon-Mann–Whitney test), and an expected allocation ratio (case:control) of 4:1, at 293 cases and 73 controls. We arrived at the final numbers of 85 control and 348 case patients, safeguarding adequate study power.

## 3. Results

A total of 348 children with T1D and 85 control subjects were enrolled in the study (182 and 44 boys, 52.3% and 51.8%, respectively), with median ages of 14.3 (IQR: 11.20–17.11) and 11.0 (8.03–14.10) years, and diabetes duration in the case group of 5.6 (3.10–8.80) years (Table 1). Only eligible subjects were recruited for the study, and no drop-out was observed due to the study’s design (single time-point measurement and medical history analysis). Participants from both groups had Fitzpatrick skin phototypes from I to III (white).

**Figure 1:**
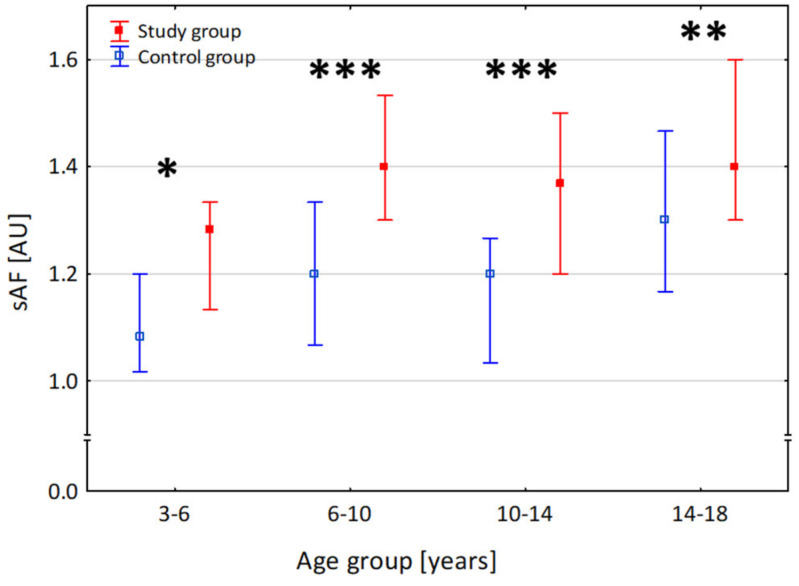
Comparison of skin autofluorescence (sAF) values across age ranges between the study group of patients with type 1 diabetes and the control group. Dots are median, and whiskers are the 25–75 centile range. * *p* < 0.05, ** *p* < 0.01, *** *p* < 0.001.

**Figure 2:**
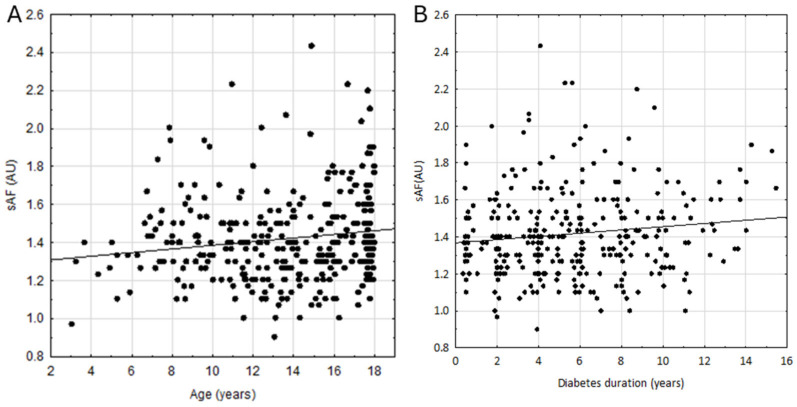
Correlation between skin autofluorescence (sAF), (**A**) patients’ age (r = 0.1487, *p* = 0.0054), and (**B**) diabetes duration (r = 0.144, *p* = 0.007).

Patients with T1D demonstrated markedly higher sAF values compared to the control group (1.40 [1.27–1.53] AU vs. 1.20 [1.07–1.30] AU; *p* = 0.004), consistent across all age groups (Figure 1, Table S1). In T1D patients, sAF positively correlated with age (r = 0.149, *p* = 0.005, Figure 2A), and we observed that sAF values were higher in girls compared to boys (1.40 [1.3–1.6] vs. 1.37 [1.27–1.50, AU], *p* = 0.011).

**Figure 3:**
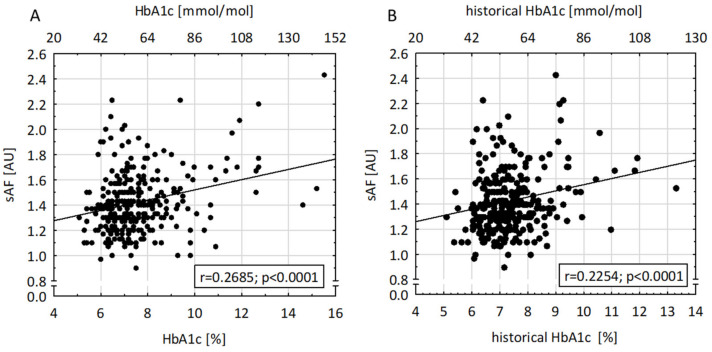
Correlation between skin autofluorescence (sAF) values, (**A**) current HbA1c (r = 0.2685, *p* < 0.0001), and (**B**) historical-HbA1c levels (i.e., average of HbA1c values measured from four months after diabetes diagnosis over the diabetes duration; r = 0.2254; *p* < 0.0001).

In T1D patients, sAF positively correlated with current and historical HbA1c concentration and diabetes duration (r = 0.269, 0.225, and 0.144; *p* < 0.0001, <0.0001, and 0.0070; Figure 3A, Figure 3B, and Figure 2B, respectively). There was no significant correlation between sAF and total daily insulin dose per kg body weight (r = 0.015, *p* = 0.795).

No significant correlation was observed between sAF and the amount of albumin excreted in urine (r = 0.108, *p* = 0.073). Because chronic microvascular complications of diabetes were found only in single patients (persistent albuminuria in two, diabetic moderate retinopathy in one, cataracts in two), differences between sAF values concerning their presence were not analyzed.

In the analyses of conventional markers of CV risk, no significant correlations were found between sAF and lipid fractions, Z-score of BMI, or the parameters from the 24 h ambulatory blood pressure monitoring (Tables S2–S4). There were no significant differences in sAF values between children with T1D based on their weight group (Figure S1). Positive correlations were found between sAF and body fat percentage (r = 0.120; *p* = 0.044) and fat-to-lean body mass ratio (FM/FFM; r = 0.132; *p* = 0.027, Table S2). sAF was positively correlated with CRP concentration (r = 0.169; *p* = 0.003, Figure S2), while a negative correlation of sAF was observed with the basal metabolic rate (BMR [kcal]; r = −0.156; *p* = 0.009, Table S2, Figure S3).

Comorbidity of T1D with celiac disease was associated with higher sAF (1.53 [1.43–1.63] vs. 1.40 [1.27–1.53, n/N = 22/348, respectively; *p* = 0.001, Figure S4). A similar effect was not present for Hashimoto’s thyroiditis (1.40 [1.27–1.60] AU vs. 1.40 AU [1.27–1.53 AU], *p* = 0.707 n/N = 22/348).

## 4. Discussion

In this study, we evaluated sAF values in a population of children and adolescents with a relatively short duration of T1D and a control group of children without glucose metabolism disorders. In all age ranges, sAF values were higher in patients with T1D than in the control group, which agrees with past works by Shah et al. and van der Heyden et al. [14,22]. Per previous works, we observed a significant positive correlation between sAF values, age, and T1D duration [13,22,23,24].

We found that girls with T1D had significantly higher sAF values than boys, which agrees with Felipe and Januszewski’s observations [24,25]. Only one paper showed no gender differences in children with T1D [25]. Higher sAF values in girls may be related to the worse glycemic control observed in girls than in boys [26,27]. The influence of diabetes on the gender difference in sAF values may be indicated by the fact that in children and adolescents without diabetes, most authors find no difference in sAF values between girls and boys and a similar result—no difference in sAF values between genders—was obtained for children and adolescents without diabetes in our previous study [19,22,25].

### 4.1. Metabolic Control

We observed a significant positive correlation between sAF and current and historical HbA1c levels, which agrees with past studies [15,25,28]. As such, sAF values depend not only on age, age-related duration of diabetes, and exposure to suboptimal glucose concentrations (low, high, and variable), but specifically reflect chronic exposure to hyperglycemia per se. This suggests that measurements of sAF could be used to assess long-term metabolic control in young patients with T1D whose previous years’ HbA1c values are unknown, or patients whose HbA1c measurements are unreliable due to additional diseases (e.g., certain types of chronic anemia). However, in other studies, the relationship between sAF and mean long-term HbA1c levels was not observed, which, according to some authors, may indicate that the measurement of sAF provides additional information independent of HbA1c and glycemic values, providing more insight into the assessment of complication risk or reflecting exposure to environmental factors, like dietary intake of AGEs or exposition to cigarette smoke [14,29,30].

### 4.2. CV Risk and Microvascular Complications

We observed no statistically significant correlation between sAF and any of the analyzed parameters of lipid metabolism (total cholesterol, LDL and HDL cholesterol concentrations, and triglycerides). It has been shown that in adults with type 1 and type 2 diabetes, higher sAF values are even more strongly associated with the risk of coronary heart disease and increased mortality than HbA1c, triglyceride, or LDL cholesterol levels [31,32,33]. In a meta-analysis by Cavero-Redondo et al., higher sAF values were a significant predictor of all-cause mortality in patients with diabetes and cardiovascular and/or renal disease [34]. However, not all studies performed in the adult population confirm the association between sAF and cardiovascular events, and participants’ characteristics, like age, may modify it (e.g., this correlation was more prominent over 35 years) [35]. Studies including pediatric populations have described correlations between sAF and proatherogenic lipid concentrations in children with T1D, including a positive correlation between sAF, total cholesterol, and triglyceride concentration and a negative correlation between sAF and HDL cholesterol concentration [13,25]. It can be assumed that the lack of significant correlations in our study was the result of relatively good lipid concentrations in the studied T1D patients and the young participants’ age. Moreover, in the publications mentioned above [13,25], the median HbA1c values were markedly higher (above 8%) than in the children participating in our study (7.30 [6.70–8.05] %). A lipid profile close to the physiological one, with the coexistence of relatively good glycemic control, may not have affected the sAF values in the study group in a significant manner.

Similarly, there were no significant associations between sAF and 24 h ABPM parameters. To date, no studies have evaluated the relationship between sAF and ABPM parameters in T1D patients. Januszewski et al. demonstrated a positive correlation between sAF and systolic, diastolic, and mean blood pressure values, as assessed by ad hoc measurements in a group of older adolescents and young adults with T1D with a relatively long duration of the disease [25]. In contrast, an analysis that included children of a similar age and duration of diabetes to those in our study did not show a relationship between sAF and random blood pressure measurements [14].

We observed no significant correlation between sAF and the z-score of BMI. Similarly, sAF values did not significantly differ between children from different weight groups. Similar results were found in other studies that included healthy and T1D pediatric patients [13,14,36]. However, we observed a significant positive correlation between sAF, body fat percentage, fat-to-lean body mass ratio (FM/FFM), and trunk body fat percentage. The above correlations could suggest that higher body fat content, especially visceral fat, may increase sAF values. Similar correlations were observed in adults with T1D, suggesting that sAF may reflect the impact of persistently high glucose concentrations on body fat content in people with T1D [37].

We also observed a negative correlation between sAF and the patients’ basic metabolic rates (BMRs). This relationship has not been described in published work to date. The relationship between BMR, body composition, and physical activity is well established [38]. Thus, the negative correlation between sAF and BMR may indirectly reflect the beneficial effect of higher physical activity on glycation severity and sAF values, i.e., lowering them [39,40,41].

Studies that evaluated the relationship between sAF and the occurrence of microvascular complications in children, adolescents, and young adults with T1D found higher sAF values in patients with retinopathy and a faster increase in sAF values in patients with microvascular complications (e.g., retinopathy or diabetic kidney disease defined as the presence of albuminuria) than in patients without complications [15,25]. We found no statistically significant relationship between sAF and urinary albumin excretion. However, the study population was only slightly affected by microvascular complications of diabetes (five cases in total), making it virtually impossible to assess the relationship between sAF and the incidence of microvascular complications in this group.

We also evaluated high-sensitivity C-reactive protein (CRP), demonstrating its significant positive correlation with sAF [42]. CRP is an acute-phase protein that reflects the severity of systemic inflammation and inhibits endothelial nitric oxide production, contributing to atherosclerotic plaque instability by increasing the expression of endothelial cell adhesion molecules, promoting monocyte recruitment to the atherosclerotic plaque, and enzymatically binding to modified LDL lipoprotein [43]. As patients with symptoms of acute infection were not included in the study group, the positive correlation between sAF and CRP may suggest an association between sAF and the severity of inflammation in the body, including the severity of chronic inflammation associated with cardiovascular risk. This relationship has not been previously explored in the literature.

### 4.3. Common Comorbidities

Taking into account, as observed in our other study (including children without diabetes), the possible influence of other diseases on sAF values, we compared sAF values between patients with the most common chronic diseases that coexist with T1D in the pediatric population: Hashimoto’s thyroiditis and celiac disease [19]. There were no differences in sAF values between patients with and without Hashimoto’s thyroiditis, but it was shown that sAF values were significantly higher in patients with T1D and concomitant celiac disease than in patients without celiac disease. In the young population with T1D (under 20 years of age), lower HDL cholesterol values were found in patients with coexisting celiac disease than in patients without it [44]. Interestingly, based on UK Biobank data, it was found that the general population of people with celiac disease had a higher risk of developing cardiovascular disease than people with no celiac disease, despite a lower prevalence of traditional cardiovascular risk factors [45]. However, since the size of the group of patients with celiac disease in our study was small (*n* = 22) and, as in the work of other authors, in adults with T1D, no correlation was found between the coexistence of celiac disease and sAF values, this association requires further studies involving a larger group of patients with celiac disease—both with and without concomitant T1D [46].

The limitation of our study is that sAF was measured at a single time point, and no longitudinal observations or repeated measurements of sAF were made in the same patient. Importantly, however, Poland is a country with low-to-moderate solar irradiance that barely reaches the skin of children’s medial forearm surface, meaning that this area does not tan, so sun exposition had hardly any chance of influencing the results of sAF measurements in the study group overall. Another limitation is that we did not gather information about participants’ dietary intake of food rich in AGEs (which could potentially affect body content of AGEs and sAF measurements), physical activity, or patients’ with celiac disease compliance with a gluten-free diet, which held us off from inquiring into the relationship and forming conclusions about the background of the observed statistical relationship of sAF with BMR and celiac disease. The strength of this study is the relatively large size of the studied group of young T1D patients and the analysis of sAF against a wide range of CV risk clinical markers, which can be considered novel.

## 5. Conclusions

In children and adolescents with T1D, sAF reflects long-term glycemic control, as assessed by mean HbA1c values over the duration of diabetes. Moreover, as sAF values are higher in children with T1D than in children without diabetes, consideration may be given to including regular sAF measurements in the overall assessment of the metabolic control of diabetes in the pediatric population, and striving to maintain or achieve sAF values characteristic for the peer population without diabetes may be a complementary goal of therapy.

In turn, in young patients with T1D of relatively short duration without coexisting microvascular complications, sAF values do not reflect cardiovascular risk, as assessed based on traditional risk factors. The negative correlation between sAF and basal metabolic rate may reflect the favorable metabolic status of individuals with lower sAF values, for example, due to their greater physical activity and higher percentage of lean body mass. This association, just like the one observed for higher sAF values in patients with coexisting T1D and celiac disease, requires further large-scale prospective studies.

## Figures and Tables

**Table 1:** Descriptive characteristics of the study and control groups.

Characteristics	T1D Group (N = 348) Median (IQR) or Number (%)	Control Group (N = 85) Median (IQR) or Number (%)
Gender (male/female)	182 (52.3%)/166 (47.7%)	45 (53%)/40 (47%)
Insulin therapy (insulin pump/MDI)	244 (70.1%)/104 (29.9%)	NA
Age [years]	14.30 (11.20–17.11)	11.01 (8.03–14.10)
Diabetes duration [years]	5.60 (3.10–8.80)	NA
Body mass index [z-score]	0.37 (−0.35–1.03)	0.08 (−0.25–0.74)
Skin autofluorescence, sAF [AU]	1.40 (1.27–1.53)	1.20 (1.07–1.30)
Daily insulin dose [units per kg per day]	0.82 (0.65–1.00)	NA
HbA1c [% and mmol/mol]	7.30 (6.70–8.05); 56.00 (50.00–64.00)	5.10 (4.90–5.20); 32.00 (30.00–33.00)
HbA1c-historical [% and mmol/mol]	7.19 (6.64–7.78); 55.00 (49.00–62.00)	NA
C-peptide [ng/dL]	0.01 (0.01–0.11)	NA
Total cholesterol [mg/dL]	167.00 (149.30–186.00)	NA
HDL cholesterol [mg/dL]	62.73 (54.66–72.22)	NA
LDL cholesterol [mg/dL]	93.00 (79.57–109.83)	NA
Triglycerides [mg/dL]	65.73 (51.36–86.85)	NA
Creatinine [mg/dL]	0.60 (0.50–0.72)	NA
CRP [mg/L]	0.40 (0.20–0.82)	NA
Total bilirubin [mg/dL]	0.57 (0.40–0.76)	NA
Albuminuria [mg/L]	7.28 (5.92–10.00)	NA

CRP—C-reactive protein; HbA1c—HbA1c at the time of sAF assessment; HbA1c-historical—mean HbA1c of all HbA1c measurements starting from the 4th month after diabetes diagnosis till sAF assessment; the median number of HbA1c measurements per patient was 11 (IQR 25–75: 3–18); MDI—multiple daily injections; NA—not applicable; T1D—type 1 diabetes.

## Data Availability

Dataset available on Zenodo (https://doi.org/10.5281/zenodo.11401389).

## Supplementary Materials

The following supporting information can be downloaded at: https://www.mdpi.com/article/10.3390/nu16121940/s1, Table S1: Comparison of skin autofluorescence (sAF) values across age ranges between the study group of patients with type 1 diabetes and the control group; Table S2: Characteristics of body composition parameters and univariate correlations between skin autofluorescence (sAF) and body composition parameters in the study group (N = 282); Table S3: Blood lipid’s fractions results and univariate correlations between skin autofluorescence (sAF) and blood lipids’ fractions in the study group (N = 348); Table S4: Ambulatory blood pressure monitoring (ABPM) results and univariate correlations between skin autofluorescence (sAF) and ABPM results in the study group (N = 196); Figure S1: Comparison of skin autofluorescence (sAF) values across age ranges between children with type 1 diabetes based on their weight group—with normal body weight (i.e., BMI 5–84th percentile, N = 253; empty circles, blue line; *p* < 0.0001 [10–14 vs. 14–18] and *p* = 0.0437 [3–6 vs. 14–18]), with overweight or obesity (i.e., BMI > 84th percentile, N = 86; filled circles, red line; *p* = 0.0001 [10–14 vs. 14–18]) and with underweight (i.e., BMI < 5th percentile, N = 8; squares, green line; no significant differences in Tukey’s post-hoc). Age and BMI group interaction *p* = 0.9767; Figure S2: Correlation between skin autofluorescence (sAF) and C-reactive protein concentration (CRP; r = 0.169, *p* = 0.0031); Figure S3: Correlation between skin autofluorescence (sAF) and basal metabolic rate (BMR; r = 0.156, *p* = 0.0089); Figure S4: sAF values in patients with type 1 diabetes and concomitant celiac disease (solid squares, N = 22) and without celiac disease (blank squares, N = 326)—comparison between the two groups (*p* = 0.001).

## References

[1] Brownlee M. (2001). Biochemistry and molecular cell biology of diabetic complications. Nature.

[2] Forbes J.M., Cooper M.E. (2013). Mechanisms of diabetic complications. Physiol. Rev..

[3] Bjornstad P., Dart A., Donaghue K.C., Dost A., Feldman E.L., Tan G.S., Wadwa R.P., Zabeen B., Marcovecchio M.L. (2022). ISPAD Clinical Practice Consensus Guidelines 2022: Microvascular and macrovascular complications in children and adolescents with diabetes. Pediatr. Diabetes.

[4] Fröhlich-Reiterer E., Elbarbary N.S., Simmons K., Buckingham B., Humayun K.N., Johannsen J., Holl R.W., Betz S., Mahmud F.H. (2022). ISPAD Clinical Practice Consensus Guidelines 2022: Other complications and associated conditions in children and adolescents with type 1 diabetes. Pediatr. Diabetes.

[5] Nathan D.M. (2014). The Diabetes Control and Complications Trial/Epidemiology of Diabetes Interventions and Complications Study at 30 Years: Overview. Diabetes Care.

[6] Bry L., Chen P.C., Sacks D.B. (2001). Effects of hemoglobin variants and chemically modified derivatives on assays for glycohemoglobin. Clin. Chem..

[7] Meerwaldt R., Graaff R., Oomen P.H.N., Links T.P., Jager J.J., Alderson N.L., Thorpe S.R., Baynes J.W., Gans R.O.B., Smit A.J. (2004). Simple non-invasive assessment of advanced glycation endproduct accumulation. Diabetologia.

[8] Sugisawa E., Miura J., Iwamoto Y., Uchigata Y. (2013). Skin autofluorescence reflects integration of past long-term glycemic control in patients with type 1 diabetes. Diabetes Care.

[9] Conway B.N., Aroda V.R., Maynard J.D., Matter N., Fernandez S., Ratner R.E., Orchard T.J. (2012). Skin intrinsic fluorescence is associated with coronary artery disease in individuals with long duration of type 1 diabetes. Diabetes Care.

[10] Monnier V.M., Sell D.R., Gao X., Genuth S.M., Lachin J.M., Bebu I. (2022). Plasma advanced glycation end products and the subsequent risk of microvascular complications in type 1 diabetes in the DCCT/EDIC. BMJ Open Diabetes Res. Care.

[11] Araszkiewicz A., Naskret D., Zozulinska-Ziolkiewicz D., Pilacinski S., Uruska A., Grzelka A., Wegner M., Wierusz-Wysocka B. (2015). Skin autofluorescence is associated with carotid intima-media thickness, diabetic microangiopathy, and long-lasting metabolic control in type 1 diabetic patients. Results from Poznan Prospective Study. Microvasc. Res..

[12] Blanc-Bisson C., Velayoudom-Cephise F.L., Cougnard-Gregoire A., Helmer C., Rajaobelina K., Delcourt C., Alexandre L., Blanco L., Mohammedi K., Monlun M. (2018). Skin autofluorescence predicts major adverse cardiovascular events in patients with type 1 diabetes: A 7-year follow-up study. Cardiovasc. Diabetol..

[13] Banser A., Naafs J.C., Hoorweg-Nijman J.J., van de Garde E.M., van der Vorst M.M. (2016). Advanced glycation end products, measured in skin, vs. HbA1c in children with type 1 diabetes mellitus. Pediatr. Diabetes.

[14] van der Heyden J.C., Birnie E., Mul D., Bovenberg S., Veeze H.J., Aanstoot H.-J. (2016). Increased skin autofluorescence of children and adolescents with type 1 diabetes despite a well-controlled HbA1c: Results from a cohort study. BMC Endocr. Disord..

[15] Cho Y.H., Craig M.E., Januszewski A.S., Benitez-Aguirre P., Hing S., Jenkins A.J., Donaghue K.C. (2017). Higher skin autofluorescence in young people with Type 1 diabetes and microvascular complications. Diabet. Med..

[16] Koetsier M., Lutgers H., de Jonge C., Links T., Smit A., Graaff R. (2010). Reference values of skin autofluorescence. Diabetes Technol. Ther..

[17] Klenovics K.S., Kollárová R., Hodosy J., Celec P., Šebeková K. (2014). Reference values of skin autofluorescence as an estimation of tissue accumulation of advanced glycation end products in a general Slovak population. Diabet. Med..

[18] Ahmad M.S., Kimhofer T., Ahmad S., AlAma M.N., Mosli H.H., Hindawi S.I., Mook-Kanamori D.O., Šebeková K., Damanhouri Z.A., Holmes E. (2017). Ethnicity and skin autofluorescence-based risk-engines for cardiovascular disease and diabetes mellitus. PLoS ONE.

[19] Jankowska M., Bobeff K., Baranowska-Jaźwiecka A., Mianowska M., Lubnauer A., Michalak A., Młynarski W., Szadkowska A., Mianowska B. (2020). Evaluation of skin autofluorescence as a surrogate of advanced glycation end products accumulation in children and adolescents with normal haemoglobin A1c values. Pediatr. Endocrinol. Diabetes Metab..

[20] Mulder D.J., Van De Water T., Lutgers H.L., Graaff R., Gans R.O., Zijlstra F., Smit A.J. (2006). Skin autofluorescence, a novel marker for glycemic and oxidative stress-derived advanced glycation endproducts: An overview of current clinical studies, evidence, and limitations. Diabetes Technol. Ther..

[21] Kułaga Z., Litwin M., Tkaczyk M., Palczewska I., Zajączkowska M., Zwolińska D., Krynicki T., Wasilewska A., Moczulska A., Morawiec-Knysak A. (2011). Polish 2010 growth references for school-aged children and adolescents. Eur. J. Pediatr..

[22] Shah S., Baez E.A., Felipe D.L., Maynard J.D., Hempe J.M., Chalew S.A. (2013). Advanced glycation endproducts in children with diabetes. J. Pediatr..

[23] Hosseini M.S., Razavi Z., Ehsani A.H., Firooz A., Afazeli S. (2021). Clinical Significance of Non-invasive Skin Autofluorescence Measurement in Patients with Diabetes: A Systematic Review and Meta-analysis. EClinicalMedicine.

[24] Felipe D.L., Hempe J.M., Liu S., Matter N., Maynard J., Linares C., Chalew S.A. (2011). Skin Intrinsic Fluorescence Is Associated with Hemoglobin A 1c and Hemoglobin Glycation Index but Not Mean Blood Glucose in Children with Type 1 Diabetes. Diabetes Care.

[25] Januszewski A.S., Xu D., Cho Y.H., Benitez-Aguirre P.Z., O’neal D.N., Craig M.E., Donaghue K.C., Jenkins A.J. (2021). Skin autofluorescence in people with type 1 diabetes and people without diabetes: An eight-decade cross-sectional study with evidence of accelerated aging and associations with complications. Diabet. Med..

[26] Samuelsson U., Anderzén J., Gudbjörnsdottir S., Steineck I., Åkesson K., Hanberger L. (2016). Teenage girls with type 1 diabetes have poorer metabolic control than boys and face more complications in early adulthood. J. Diabetes Complicat..

[27] Jarosz-Chobot P., Polańska J., Myśliwiec M., Szadkowska A., Fendler W., Kamińska H., Chumiecki M., Mianowska B., Techmańska I., Sztangierska B. (2012). PolPeDiab study group. Multicenter cross-sectional analysis of values of glycated haemoglobin (HbA1c) in Polish children and adolescents with long-term type 1 diabetes in Poland: PolPeDiab study group. Pediatr. Endocrinol. Diabetes Metab..

[28] Báez E.A., Shah S., Felipe D., Maynard J., Lefevre S., Chalew S.A. (2015). Skin advanced glycation endproducts are elevated at onset of type 1 diabetes in youth. J. Pediatr. Endocrinol. Metab..

[29] Barat P., Cammas B., Lacoste A., Harambat J., Vautier V., Nacka F., Corcuff J.-B. (2012). Advanced glycation end products in children with type 1 diabetes: Family matters?. Diabetes Care.

[30] Reurean-Pintilei D., Stoian A.P., Potcovaru C.-G., Salmen T., Cinteză D., Stoica R.-A., Lazăr S., Timar B. (2024). Skin Autofluorescence as a Potential Adjunctive Marker for Cardiovascular Risk Assessment in Type 2 Diabetes: A Systematic Review. Int. J. Mol. Sci..

[31] Meerwaldt R., Lutgers H.L., Links T.P., Graaff R., Baynes J.W., Gans R.O., Smit A.J. (2007). Skin autofluorescence is a strong predictor of cardiac mortality in diabetes. Diabetes Care.

[32] Mulder D.J., van Haelst P.L., Gross S., de Leeuw K., Bijzet J., Graaff R., Gans R.O., Zijlstra F., Smit A.J. (2008). Skin autofluorescence is elevated in patients with stable coronary artery disease and is associated with serum levels of neopterin and the soluble receptor for advanced glycation end products. Atherosclerosis.

[33] Mulder D.J., van Haelst P.L., Graaff R., Gans R.O., Zijlstra F., Smit A.J. (2009). Skin autofluorescence is elevated in acute myocardial infarction and is associated with the one-year incidence of major adverse cardiac events. Neth. Heart J..

[34] Cavero-Redondo I., Soriano-Cano A., Álvarez-Bueno C., Cunha P.G., Martínez-Hortelano J.A., Garrido-Miguel M., Berlanga-Macías C., Martínez-Vizcaíno V. (2018). Skin autofluorescence–indicated advanced glycation end products as predictors of cardiovascular and all-cause mortality in high-risk subjects: A systematic review and meta-analysis. J. Am. Heart Assoc..

[35] van Waateringe R.P., Fokkens B.T., Slagter S.N., van der Klauw M.M., van Vliet-Ostaptchouk J.V., Graaff R., Paterson A.D., Smit A.J., Lutgers H.L., Wolffenbuttel B.H.R. (2019). Skin autofluorescence predicts incident type 2 diabetes, cardiovascular disease and mortality in the general population. Diabetologia.

[36] Lentferink Y.E., Van Teeseling L., Knibbe C.A.J., Van Der Vorst M.M.J. (2019). Skin autofluorescence in children with and without obesity. J. Pediatr. Endocrinol. Metab..

[37] Zawada A., Naskret D., Niedźwiecki P., Grzymisławski M., Zozulińska-Ziółkiewicz D., Dobrowolska A. (2020). Excess body fat increases the accumulation of advanced glycation end products in the skin of patients with type 1 diabetes. Adv. Clin. Exp. Med..

[38] Speakman J.R., Selman C. (2003). Physical activity and resting metabolic rate. Proc. Nutr. Soc..

[39] Köchli S., Endes K., Trinkler M., Mondoux M., Zahner L., Hanssen H. (2020). Association of physical fitness with skin autofluorescence-derived advanced glycation end products in children. Pediatr. Res..

[40] Hjerrild J.N., Wobbe A., Stausholm M.B., Larsen A.E., Josefsen C.O., Malmgaard-Clausen N.M., Dela F., Kjaer M., Magnusson S.P., Hansen M. (2019). Effects of long-term physical activity and diet on skin glycation and Achilles tendon structure. Nutrients.

[41] Hirai T., Fujiyoshi K., Yamada S., Matsumoto T., Kikuchi J., Ishida K., Ishida M., Yamaoka-Tojo M., Inomata T., Shigeta K. (2022). Advanced Glycation End Products Are Associated with Diabetes Status and Physical Functions in Patients with Cardiovascular Disease. Nutrients.

[42] The Emerging Risk Factors Collaboration (2012). C-Reactive Protein, Fibrinogen, and Cardiovascular Disease Prediction. N. Engl. J. Med..

[43] Badimon L., Peña E., Arderiu G., Padró T., Slevin M., Vilahur G., Chiva-Blanch G. (2018). C-reactive protein in atherothrombosis and angiogenesis. Front. Immunol..

[44] Warncke K., Liptay S., Fröhlich-Reiterer E., Scheuing N., Schebek M., Wolf J., Rohrer T.R., Meissner T., Holl R.W. (2016). Vascular risk factors in children, adolescents, and young adults with type 1 diabetes complicated by celiac disease: Results from the DPV initiative. Pediatr. Diabetes.

[45] Conroy M., Allen N., Lacey B., Soilleux E., Littlejohns T. (2023). Association between coeliac disease and cardiovascular disease: Prospective analysis of UK Biobank data. BMJ Med..

[46] Bakker S., Tushuizen M., Gözütok E., Çiftci A., Gelderman K., Mulder C., Simsek S. (2015). Advanced glycation end products (AGEs) and the soluble receptor for AGE (sRAGE) in patients with type 1 diabetes and coeliac disease. Nutr. Metab. Cardiovasc. Dis..

