# Supplementary material for: Assessment of Skin Autofluorescence and Its Association with Glycated Hemoglobin, Cardiovascular Risk Markers, and Concomitant Chronic Diseases in Children with Type 1 Diabetes
Source: Nutrients. 2024 Jun 19;16(12):1940. doi: 10.3390/nu16121940 (PMC11206751; doi:10.3390/nu16121940)
Supplement: Supplementary file 1 [file nutrients-16-01940-s001.zip › nutrients-3060469-supplementary.pdf]

nutrients

Supplementary Materials

Assessment of Skin Autofluorescence and Its Association with Glycated Hemoglobin, Cardiovascular Risk Markers, and Concomitant Chronic Diseases in Children with Type 1 Diabetes

Marta Jankowska [1,2], Agnieszka Szadkowska [1], Iwona Pietrzak [1], Jędrzej Chrzanowski [3], Julia Sołek [4], Wojciech Fendler [3] and Beata Mianowska [1,*]

1 Department of Pediatrics, Diabetology, Endocrinology and Nephrology, Medical University of Lodz, 91-738 Lodz, Poland; marta.jankowska87@gmail.com (M.J.); agnieszka.szadkowska@umed.lodz.pl (A.S.); iwona.pietrzak@umed.lodz.pl (I.P.);
2 Department of Developmental Neurology and Epileptology, Polish Mother's Memorial Hospital-Research Institute, 93-338 Lodz, Poland
3 Department of Biostatistics and Translational Medicine, Medical University of Lodz, 92-215 Lodz, Poland; jedrzej.chrzanowski@umed.lodz.pl (J.C.); wojciech.fendler@umed.lodz.pl (W.F.)
4 Department of Pathology, Chair of Oncology, Medical University of Lodz, 92-213 Lodz, Poland; juliasolek@gmail.com
* Correspondence: beata.mianowska@umed.lodz.pl; Tel.: +48-42-6177750

Table S1. Comparison of skin autofluorescence (sAF) values across age ranges between the study group of patients with type 1 diabetes and the control group.

Age Range [years]	Skin Autofluorescence, sAF [AU]				p
	Patients with Type 1 Diabetes (N = 348)		Control Group (N = 85)		
	N	Median (IQR)	N	Median (IQR)	
3–6	10	1.28 (1.13–1.33)	16	1.08 (1.02–1.20)	0.014
6–10	55	1.40 (1.30–1.53)	20	1.20 (1.07–1.33)	<0.001
10–14	99	1.37 (1.20–1.50)	27	1.20 (1.03–1.27)	<0.001
14–18	184	1.40 (1.30–1.60)	22	1.30 (1.17–1.47)	0.009

Table S2. Characteristics of body composition parameters and univariate correlations between skin autofluorescence (sAF) and body composition parameters in the study group (N = 282).

Characteristics	Median (IQR)	r	p
**Fat mass, FM [kg]**	12.20 (8.00–17.40)	0.050	0.4060
**Body fat percentage [%]**	22.45 (18.40–27.90)	0.120	0.0440
**Fat free mass, FFM [kg]**	43.20 (32.00–51.90)	−0.105	0.0770
**Total body water [kg]**	31.60 (23.40–38.00)	−0.097	0.1020
**Bone tissue mass [kg]**	2.20 (1.70–2.60)	−0.122	0.0450
**Basal metabolic rate, BMR [kcal]**	1473.50 (1292.00–1786.00)	−0.156	0.0090
**Fat mass of trunk [kg]**	5.40 (3.50–7.80)	0.054	0.3630
**Trunk body fat percentage [%]**	17.80 (14.30–23.00)	0.112	0.0600
**Fat mass of trunk/body weight [kg/kg]**	0.10 (0.08–0.13)	0.134	0.0240
**FFM/body weight [kg/kg]**	0.77 (0.72–0.82)	−0.096	0.1090
**FM/FFM [kg/kg]**	0.29 (0.23–0.34)	0.132	0.0270
**Z-score BMI (kg/m^2)**	0.37 (−0.35–1.03)	−0,086	0.1030

Table S3. Blood lipid's fractions results and univariate correlations between skin autofluorescence (sAF) and blood lipids' fractions in the study group (N = 348).

Characteristics	Median (IQR)	r	p
**Total cholesterol [mg/dL]**	167.00 (149.30–186.00)	0.046	0.4000
**HDL cholesterol [mg/dL]**	62.73 (54.66–72.22)	−0.015	0.7800
**LDL cholesterol [mg/dL]**	93.00 (79.57–109.83)	0.063	0.2530
**Triglycerides [mg/dL]**	65.73 (51.36–86.85)	0.078	0.1510

Table S4. Ambulatory blood pressure monitoring (ABPM) results and univariate correlations between skin autofluorescence (sAF) and ABPM results in the study group (n = 196).

Blood pressure (BP) characteristics	Median (IQR)	r	p
**Mean systolic BP - daytime [mmHg]**	115.50 (110.00–121.00)	−0.144	0.0430
**Mean diastolic BP - daytime [mmHg]**	70.00 (67.00–74.00)	0.069	0.3390
**Mean heart rate - daytime [BPM]**	84.00 (78.00–91.00)	0.065	0.3640
**Systolic BP measurements >95th percentile - daytime [%]**	5.00 (0.00–16.40)	−0.057	0.4260
**Diastolic BP measurements >95th percentile - daytime [%]**	6.00 (2.00–14.90)	0.042	0.5630
**Mean systolic BP – nighttime [mmHg]**	101.00 (97.00–110.00)	−0.118	0.1020
**Mean diastolic BP – nighttime [mmHg]**	57.00 (54.00–60.00)	0.112	0.1210
**Mean heart rate - nighttime [BPM]**	69.00 (61.00–73.00)	0.054	0.4560
**Systolic BP measurements >95th percentile – nighttime [%]**	4.30 (0.00–14.80)	−0.051	0.4770
**Diastolic BP measurements >95th percentile – nighttime [%]**	7.70 (0.00–18.50)	−0.016	0.8260
**Systolic BP dipping [%]**	11.80 (9.00–14.20)	−0.057	0.4320
**Diastolic BP dipping [%]**	17.20 (13.60–21.90)	−0.044	0.5450

BP, blood pressure; 95.percentile—95th percentile of BP value for sex and height. BP dipping, states by which percent the mean nighttime BP is lower than the mean daytime BP. BPM—beats per minute.

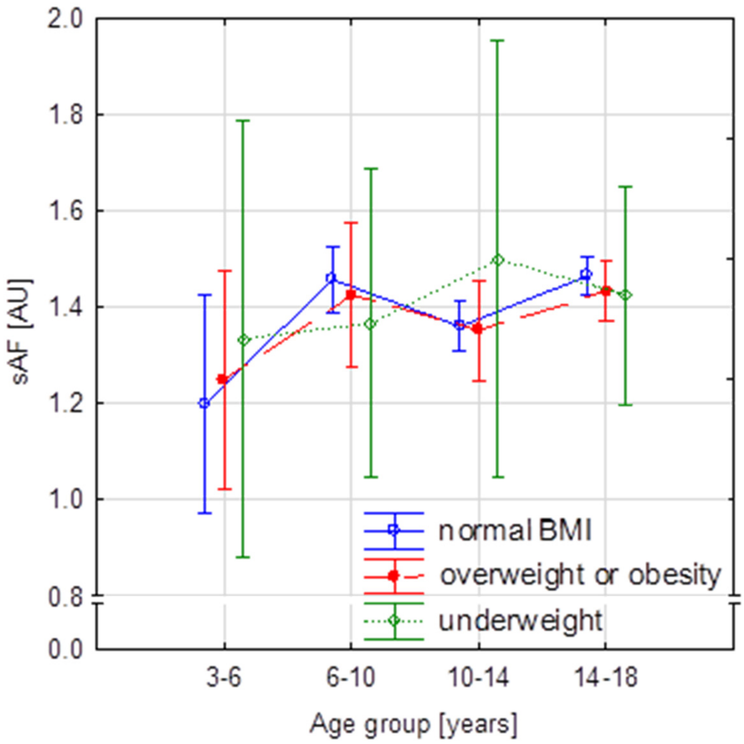

Figure S1. Comparison of skin autofluorescence (sAF) values across age ranges between children with type 1 diabetes based on their weight group—with normal body weight (i.e., BMI 5–84th percentile, n = 253; empty circles, blue line; p < 0.0001 [10–14 vs. 14–18] and p = 0.0437 [3–6 vs. 14–18]), with overweight or obesity (i.e., BMI > 84th percentile, N = 86; filled circles, red line; p = 0.0001 [10–14 vs. 14–18]) and with underweight (i.e., BMI < 5th percentile, N = 8; squares, green line; no significant differences in Tukey's post-hoc). Age and BMI group interaction p = 0.9767.

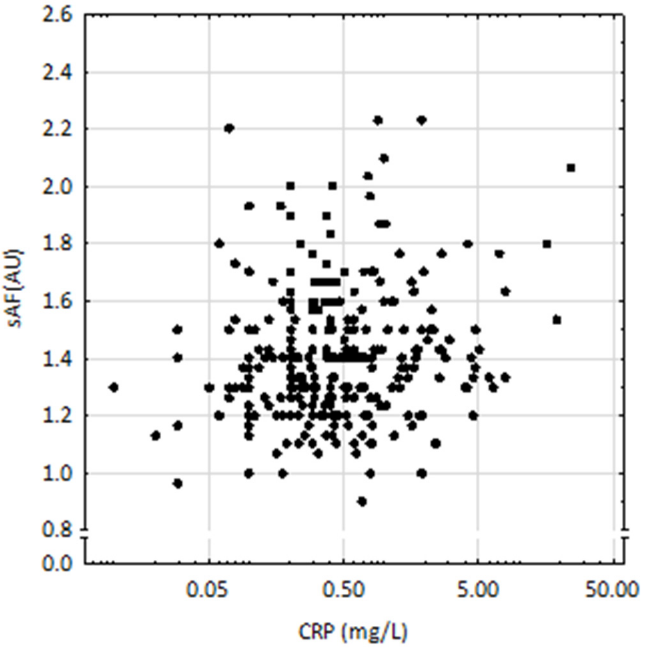

Figure S2. Correlation between skin autofluorescence (sAF) and C-reactive protein concentration (CRP; r = 0.169, *p* = 0.0031).

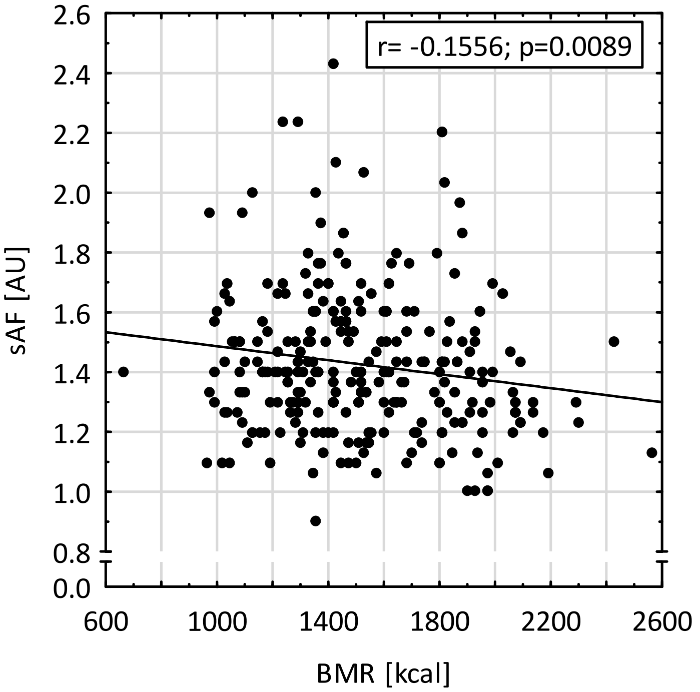

Figure S3. Correlation between skin autofluorescence (sAF) and basal metabolic rate (BMR; r = 0.156, *p* = 0.0089).

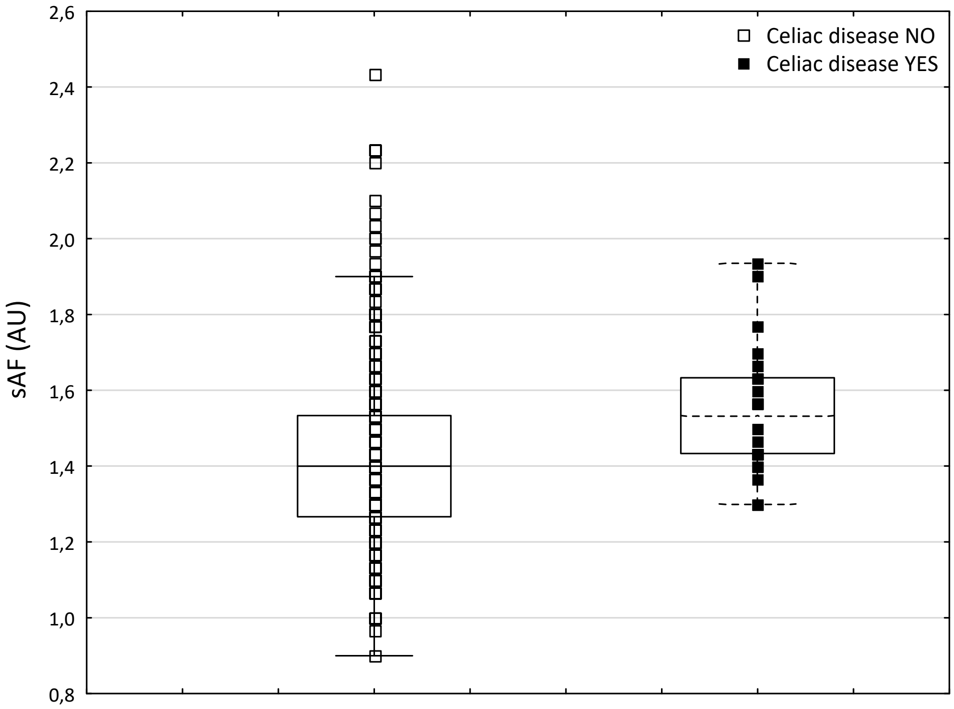

Figure S4. sAF values in patients with type 1 diabetes and concomitant celiac disease (solid squares, N = 22) and without celiac disease (blank squares, N = 326)—comparison between the two groups ($p = 0.001$).
